# Data on expression of lipoxygenases-5 and -12 in the normal and acetaminophen-damaged liver

**DOI:** 10.1016/j.dib.2016.03.079

**Published:** 2016-03-31

**Authors:** Maria Suciu, Alexandra T. Gruia, Dragos V. Nica, Seyed M.R. Azghadi, Ani A. Mic, Felix A. Mic

**Affiliations:** a Electron Microscopy Integrated Laboratory, National Institute for Research and Development of Isotopic and Molecular Technologies, 67-103 Donath St., 400293 Cluj-Napoca, Romania; b Molecular Biology and Biotechnology Department, Faculty of Biology and Geology, Babeş-Bolyai University, Cluj 5-7 Clinicilor St., 400006 Napoca, Romania; c Department of Functional Sciences, University of Medicine and Pharmacy “Victor Babes”, 2 Eftimie Murgu Sq., 300041 Timisoara, Romania; d Regional Center for Transplant Immunology, County Clinical Emergency Hospital Timisoara, 10 Iosif Bulbuca Blvd., 300736 Timisoara, Romania; e Faculty of Animal Sciences and Biotechnologies, Banat׳s University of Agricultural Sciences and Veterinary Medicine “King Michael I of Romania”, Timisoara, Romania

## Abstract

Here we present additional data on the expression of lipoxygenases -5 and -12 in the normal and acetaminophen-damaged liver, which are associated with our manuscript recently published in Chemico-Biological Interactions on lipid metabolism and eicosanoid signaling pathways involved in acetaminophen-induced liver damage in a mouse model (http://dx.doi.org/10.1016/j.cbi.2015.10.019 [1]). It has been demonstrated that the expression of lipoxygenase-5 and leukotriene formation are increased in the livers of rats with carbon tetrachloride (CCl_4_)-induced cirrhosis (http://dx.doi.org/10.1053/gast.2000.17831 [2]). In addition, the lipoxygenase-12 is known to be expressed in the resident macrophage population of the liver (http://dx.doi.org/10.1016/S0014-5793(99)00396-8 [3]).

Mice were injected with acetaminophen, and at 48 h their livers were processed for immunohistochemistry with anti-mouse lipoxygenase-5 and -12 antibodies. At the same time point, the RNA was also extracted from the liver to assess the expression of lipoxygenase-5 and -12 genes via qPCR analysis. Our results show that lipoxygenase-5 expression, but not that of lipoxygenase-12, changes significantly in the acetominophen-damaged liver.

**Specifications Table**

**Table:** 

Subject area	*Biology*
More specific subject area	*Toxicology*
Type of data	*Graph, figure*
How data was acquired	*Microscopy, qPCR*
Data format	*Analyzed*
Experimental factors	*Mice were injected with a dose of 400 mg/kg acetaminophen; Immunohistochemistry qPCR*
Experimental features	*Mice were injected with a dose of 400 mg/kg acetaminophen and liver samples were collected at 48 h post-administration. The tissue samples were processed for immunohistochemistry for lipoxygenase-5 and -12 and the RNA was extracted for qPCR analysis of the corresponding genes.*
Data source location	*Timisoara, Romania*
Data accessibility	*Data is within this article*

## Value of the data

• This data set provides further evidence for the involvement of the eicosanoid signaling pathway in the mechanism of acetaminophen-induced liver damage.
• The data show that immunohistochemical and gene expression of lipoxygenase-5 and -12 are enhanced in the liver tissue during acetaminophen-induced damage.
• These data show that both lipoxygenase-5 and -12 signaling pathways are activated during acetaminophen-induced liver damage.
• These data are valuable to researchers interested in the molecular background of eicosanoid signaling pathway during acetaminophen-induced liver damage.

## 1 Data

**Fig. 1:**
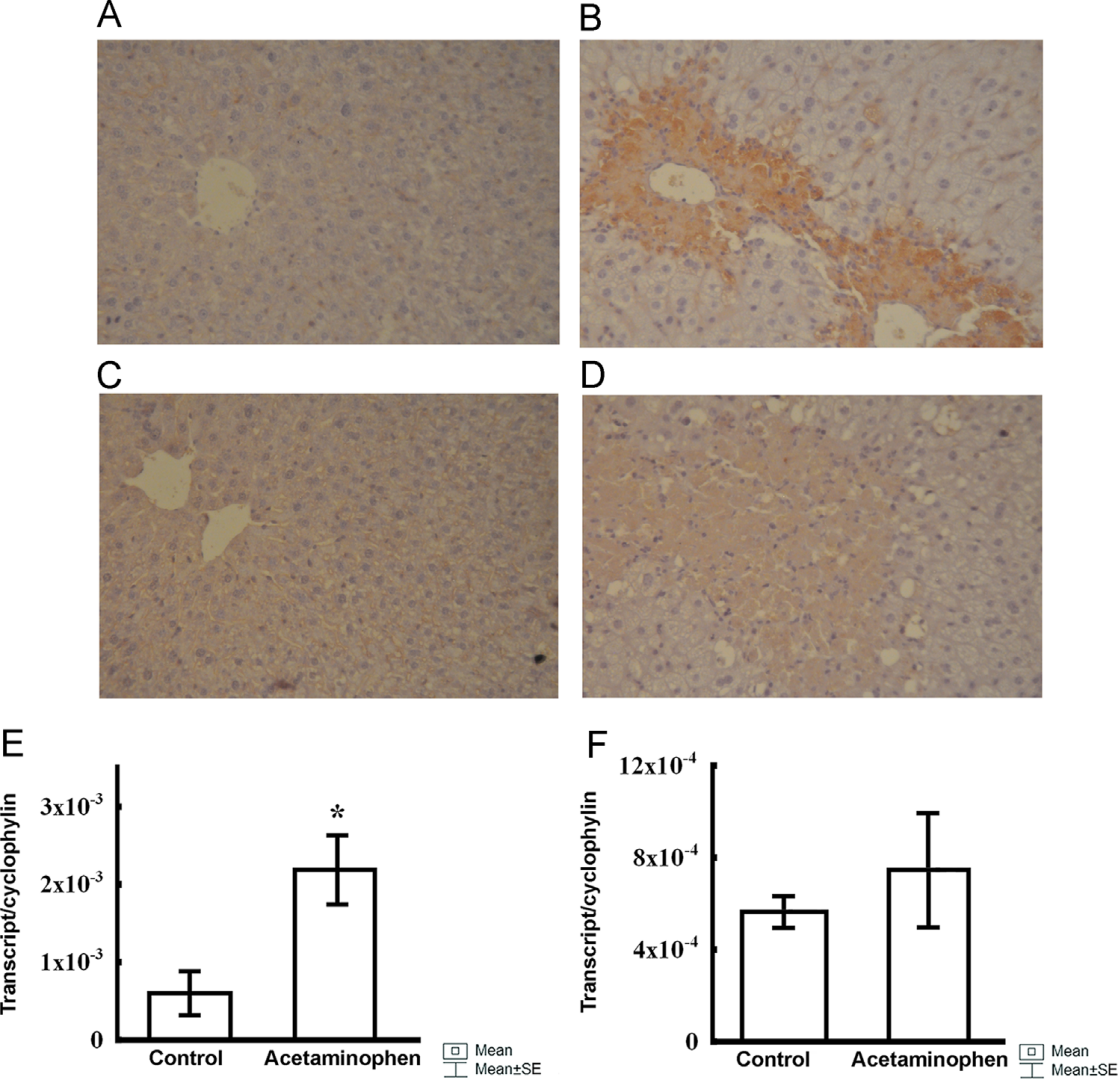
The immunohistochemical expression of lipoxygenase-5 at 48 h in the control liver (A, 40× magnification) and acetaminophen-induced liver injury (B, 40× magnification). The immunohistochemical expression of liver lipoxygenase-12 at 48 h in controls (C, 40× magnification) and acetaminophen-injected specimens (D, 40× magnification). Real-time PCR expression of lipoxygenase-5 gene at 48 h in the control liver and acetaminophen-induced liver injury (E). Real-time PCR expression of lipoxygenase-12 gene in control and acetaminophen-injected liver specimens at 48 h (F).

Here we present immunohistochemical and qPCR data showing that lipoxygenases-5 and -12 are also activated during acetaminophen-induced liver damage[1]. The lipoxygenase-5 appeared to be weakly expressed in the normal liver parenchyma (Fig. 1A, 40× magnification), but at 48 h acetaminophen administration consistently enhanced its expression in the damaged livers[2], primarily around the centrilobular veins (Fig. 1B, 40× magnification).

The lipoxygenase-12 also appeared to stain weakly the normal liver parenchyma (Fig. 1C, 40× magnification), whereas acetaminophen administration enhanced, but only modestly, its expression at 48 h (Fig. 1D, 40× magnification), especially around the centrilobular veins[3]. The expression pattern of lipoxygenase-12 overlapped with that observed for the lipoxygenase-5 in the damaged liver.

When the expression of lipoxygenase-5 and -12 was examined in the control and acetaminophen-treated livers, we found that at 48 h, the expression of both lipoxygenases was up-regulated, but significant differences were found only for lipoxygenase-5 (Fig. 1E and F).

## 2 Experimental design, materials and methods

### 2.1 Immunohistochemistry

The liver samples were fixed overnight in 4% formaldehyde, embedded in paraffin, sectioned at a 4 µm thickness, and then mounted on silanized glass slides. LSAB2 kits from Dako Denmark A/S (Glostrup, Denmark) were used for immunochemical detection of acetaminophen-induced cellular damage. After de-paraffination and rehydration, the slides were incubated with 3% hydrogen peroxide solution for 5 min. Non-specific binding of IgG was blocked using 1% bovine serum albumin in phosphate buffer saline for half an hour. The slides were then incubated overnight using the following primary antibodies: goat anti-rat anti-LOX-5, and rabbit anti-rat anti-LOX-12, both at 1:250 dilution. Following incubation with biotin-labeled secondary antibody for 30 min, streptavidin–horse radish peroxidase was added for another 30 min, and the slides were stained with a 3,3׳-diaminobenzidine chromogen solution (http://dx.doi.org/10.4049/jimmunol.181.11.8027 [4]). The slides were counterstained with hematoxylin, dehydrated, mounted, and photographed. For negative controls, the primary antibody was omitted from the procedure.

### 2.2 qPCR

We extracted RNA from the acetaminophen-treated livers after the blood was flushed off from the liver by intra-ventricular perfusion with PBS. Liver samples collected from untreated mice (at 48 h post vehicle), as well as from acetaminophen-injected animals (at 48 h post-treatment) were analyzed to quantify the mRNA expression of lipoxygenase-5 and -12 on a LightCycler 480 RT-PCR instrument, with cyclophilin A being used as a housekeeping gene. The qPCR analysis for lipoxygenase-5, -12, and cyclophilin A was performed according to primer sequences and PCR conditions previously published (http://dx.doi.org/10.1371/journal.pone.0011979 [5], http://dx.doi.org/10.1186/1476-069X-5-24 [6]). The quantification of gene expression was based on the Ct value for each sample. The Ct values were calculated as the average of duplicate measurements, and the data obtained were normalized to the endogenous housekeeping gene cyclophilin A.

## 3 Statistical analysis

The data are displayed as mean (*X*)±SE from at least three independent experiments. Statistical analysis was performed with the statistical package Gnumeric Spreadsheet (Gnome Foundation, Orinda, CA, USA). Planned pairwise comparisons between groups were performed by using Student׳s *t*-tests; *p<*0.05 was the criterion of significance.
