# Supplementary material for: Data on expression of lipoxygenases-5 and -12 in the normal and acetaminophen-damaged liver
Source: Data Brief. 2016 Mar 31;7:1199–203. doi: 10.1016/j.dib.2016.03.079 (PMC4927949; doi:10.1016/j.dib.2016.03.079)
Supplement: Supplementary file 1 — Supplementary material [file mmc1.doc]

Conflict of Interest Form

We confirm that the manuscript has been read and approved by all named authors and that there are no other persons who satisfied the criteria for authorship but are not listed. We further confirm that the order of authors listed in the manuscript has been approved by all of us. We have no other conflicts of interest to declare.

Dragos Nica, corresponding author
